# Hispanic and Latino Dementia Caregiver Coping and Associations With Daily Stress, Depression, and Anxiety

**DOI:** 10.1093/geroni/igaf122.1704

**Published:** 2025-12-31

**Authors:** Nury Rodriguez Colmenares, Natashia Bibriescas, Frank Puga

**Affiliations:** The University of Alabama at Birmingham, Birmingham, Alabama, United States; University of Alabama at Birmingham, Birmingham, Alabama, United States; University of Alabama at Birmingham, Birmingham, Alabama, United States

## Abstract

Hispanic and Latino (H&L) caregivers of people living with dementia experience significant emotional and psychological strain, yet little is known about how their coping strategies influence daily mental health. This study examined coping strategies used by H&L caregivers and associations with daily stress, depression, and anxiety symptoms. Data from 166 participants were analyzed using multiple linear regression. Over 21 days, caregivers completed daily surveys assessing coping strategies (Brief-COPE inventory), depressive and anxiety symptoms (PROMIS Emotional Distress Short Form Depression and Anxiety), and stress related to behavioral symptoms (adapted Neuropsychiatric Inventory-Questionnaire). Most caregivers identified as female (87.3%), with a mean age of 57 years (SD = 9.7), and were adult children caring for a biological parent (62.7%). The depression model was statistically significant, with dysfunctional coping predicting higher depression symptoms (β = 1.06, SE = 0.09, t = 10.80, p < 0.001). Similarly, the anxiety model was significant, with dysfunctional coping predicting higher anxiety symptoms (β = 1.00, SE = 0.10, t = 9.46, p < 0.001). The stress model was also significant with both dysfunctional coping (β = 1.02, SE = 0.51, t = 1.99, p < 0.05) and providing care for someone other than a parent or a spouse (β = 7.27, SE = 3.27, t = 2.22, p < 0.05) predicting higher stress levels. These findings highlight how dysfunctional coping influences caregiver well-being and underscore the need for interventions that promote adaptive coping strategies among H&L dementia caregivers.

